# Encoding of Demographic and Anatomical Information in Chest X-Ray-Based Severe Left Ventricular Hypertrophy Classifiers

**DOI:** 10.3390/biomedicines13092140

**Published:** 2025-09-02

**Authors:** Basudha Pal, Rama Chellappa, Muhammad Umair

**Affiliations:** 1 Department of Electrical and Computer Engineering, Johns Hopkins University, Baltimore, MD 21218, USA; rchella4@jhu.edu; 2 Department of Biomedical Engineering, Johns Hopkins University, Baltimore, MD 21218, USA; 3 The Russell H. Morgan Department of Radiology and Radiological Sciences, The Johns Hopkins Hospital, Baltimore, MD 21287, USA; mu2331@cumc.columbia.edu; 4 Department of Radiology, Columbia University Irving Medical Center, New York, NY 10032, USA

**Keywords:** deep learning, Chest X-Rays, echocardiographic phenotypes, mutual information, interpretability

## Abstract

**Background.** Severe left ventricular hypertrophy (SLVH) is a high-risk structural cardiac abnormality associated with increased risk of heart failure. It is typically assessed using echocardiography or cardiac magnetic resonance imaging, but these modalities are limited by cost, accessibility, and workflow burden. We introduce a deep learning framework that classifies SLVH directly from chest radiographs, without intermediate anatomical estimation models or demographic inputs. A key contribution of this work lies in interpretability. We quantify how clinically relevant attributes are encoded within internal representations, enabling transparent model evaluation and integration into AI-assisted workflows. **Methods.** We construct class-balanced subsets from the CheXchoNet dataset with equal numbers of SLVH-positive and negative cases while preserving the original train, validation, and test proportions. ResNet-18 is fine-tuned from ImageNet weights, and a Vision Transformer (ViT) encoder is pretrained via masked autoencoding with a trainable classification head. No anatomical or demographic inputs are used during training. We apply Mutual Information Neural Estimation (MINE) to quantify dependence between learned features and five attributes: age, sex, interventricular septal diameter (IVSDd), posterior wall diameter (LVPWDd), and internal diameter (LVIDd). **Results.** ViT achieves an AUROC of 0.82 [95% CI: 0.78–0.85] and an AUPRC of 0.80 [95% CI: 0.76–0.85], indicating strong performance in SLVH detection from chest radiographs. MINE reveals clinically coherent attribute encoding in learned features: age > sex > IVSDd > LVPWDd > LVIDd. **Conclusions.** This study shows that SLVH can be accurately classified from chest radiographs alone. The framework combines diagnostic performance with quantitative interpretability, supporting reliable deployment in triage and decision support.

## 1. Introduction

Early detection of left ventricular (LV) abnormalities such as severe hypertrophy (SLVH) is essential for cardiovascular risk management [1]. Conventionally, echocardiography remains the clinical standard for assessing LV structure [2]. However, its use is typically confined to individuals with a high pretest probability, in part due to reliance on specialized equipment and operator expertise [3]. While cardiac magnetic resonance imaging (MRI) provides accurate and reproducible assessments of myocardial structure and function [4], its cost, limited availability, and scanning time restrict routine population-level screening. Chest X-rays (CXRs) are widely available in clinical practice, noninvasive, inexpensive, and commonly used as a first-line imaging modality [5]. Although CXRs lack three-dimensional detail and dynamic imaging capabilities for comprehensive cardiac anatomical assessment, recent deep learning models have demonstrated their ability to extract clinically meaningful information and estimate echocardiographic indices such as IVSDd, LVIDd, and LVPWDd [6].

However, directly classifying SLVH from CXRs remains challenging due to the subtle nature of phenotypic changes and 3-dimensional overlap of the modality. Consequently, prior work [6] adopts an indirect approach of first performing regression to estimate intermediate anatomical variables such as IVSDd, LVIDd, and LVPWDd, followed by thresholding to determine SLVH status. Additionally, since clinical thresholds for defining SLVH depend on age and gender, demographic information is explicitly provided to guide a learnable thresholding process and improve predictive alignment with clinical guidelines [7]. While this strategy can improve predictive performance, it also introduces architectural and conceptual drawbacks. First, it creates vulnerability to error cascading, where inaccuracies in anatomical regression propagate through the model and compromise final classification. Second, explicitly including demographic variables can result in confounding, as these attributes may serve as proxies for the outcome rather than truly independent predictors, thus reducing model transparency and interpretability. These issues make it harder to understand model behavior and raise concerns for clinical deployment, where clarity and robustness are essential. Beyond the risk of error propagation and confounding, from a representation learning perspective, it is preferable to train models directly on the end task [8]. A dedicated classification model should learn features for discriminating between SLVH-positive and SLVH-negative cases, potentially offering better alignment with the final diagnostic objective and fewer sources of modeling error while implicitly encoding relevant clinical and demographic attributes.

**Figure 1:**
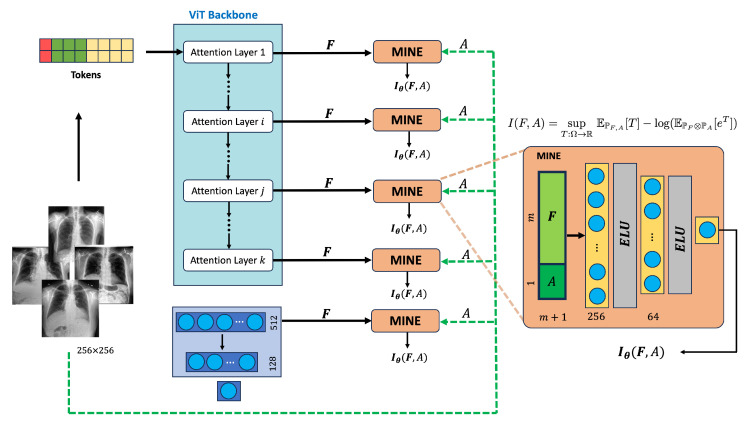
MINE-based expressivity pipeline for a ViT backbone used in SLVH classification. Feature representations $F\in R^{m}$ are extracted from a designated transformer layer and concatenated with scalar attribute values *A* (such as age, sex, IVSDd, LVPWd, or LVIDd), resulting in (m+1)-dimensional inputs. These inputs are passed through a two-layer multilayer perceptron (MLP) within the MINE module, which is trained to estimate mutual information and quantify the degree to which each attribute is encoded in the model’s internal features.

In this work, we propose a simplified yet effective alternative. We introduce a direct classification framework that predicts SLVH status (present or absent) from chest X-rays alone, without relying on intermediate structural predictions or demographic inputs. In this way, we avoid the risks associated with explicitly including anatomical or demographic variables, such as confounding and error propagation, while still aiming for a model that captures clinically meaningful patterns. To verify that our classifier remains aligned with relevant clinical attributes despite not receiving them as inputs, we apply Mutual Information Neural Estimation (MINE) [9] to quantify the relationship between internal feature representations and relevant attributes such as age, sex, IVSDd, LVIDd, and LVPWDd. MINE estimates mutual information by training a neural network discriminator to distinguish joint from independent samples using the Donsker–Varadhan representation [10], making it tractable in high-dimensional settings. This framework has been used to reveal how sensitive attributes are encoded in domains such as face recognition [11] and person reidentification [12]. We adapt it here to examine how clinical and demographic information is encoded across early, mid, and late layers in SLVH classifiers trained without access to those attributes. The resulting score, which we refer to as expressivity, reflects the degree to which each attribute is entangled in the learned representation. Figure 1 shows the overall block diagram of our proposed approach.

This analysis quantifies the extent to which internal feature representations encode key variables such as age, sex, and anatomical measurements like IVSDd and LVPWDd. Although these factors are not part of the model input, they are strongly associated with SLVH and should be reflected in the features learned by an effective model. MINE helps us confirm that the model attends to this underlying structure implicitly, supporting both interpretability and clinical trust without compromising design efficiency or robustness. Our contributions are as follows:

- Modeling: We present a direct SLVH classification framework using both convolutional as well as transformer backbones with only chest X-ray images, removing reliance on anatomical regressors and demographic inputs. This improves generalizability and streamlines model design.
- Evaluation: We address limitations in prior work by constructing a balanced subset of the CheXchoNet dataset, enabling improved discriminative performance and reliable benchmarking. Performance is assessed using AUROC and AUPRC.
- Interpretability: We apply MINE to estimate mutual information between internal features and clinical attributes, enabling quantitative analysis of attribute encoding without requiring explicit supervision. This supports a more interpretable and clinically aligned deployment of deep learning models.

To our knowledge, this is the first study to propose a direct classification framework for detecting SLVH from chest X-rays, bypassing the need for intermediate anatomical modeling or demographic inputs. Moreover, we are the first to introduce MINE as a tool for analyzing internal representations in deep learning based cardiac imaging. Beyond the specific application to SLVH classification, MINE represents a generalizable framework for quantifying feature–attribute relationships in deep learning models. Its ability to uncover clinically meaningful correlations makes it particularly well suited for advancing interpretability in cardiac imaging and other domains of clinical computer vision.

## 2. Materials and Methods

### 2.1. Dataset and Preprocessing

**Figure 2:**
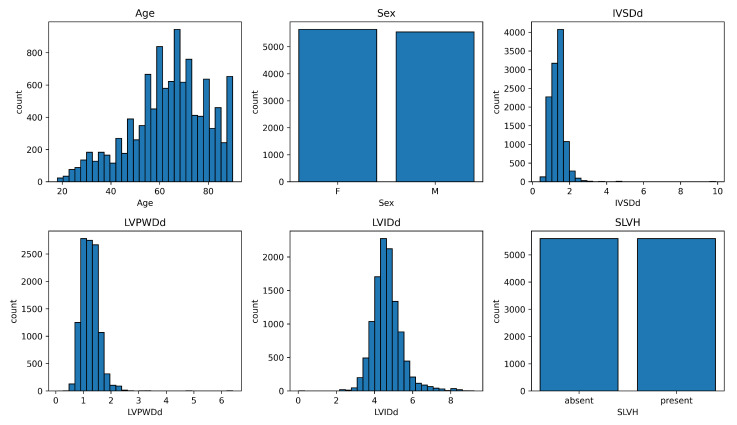
Data distribution for training data of the balanced dataset.

We use the CheXchoNet dataset [6], which pairs CXRs with echocardiography-derived structural measurements. To mitigate the class imbalance that hindered previous regression-based approaches [6], we construct class-balanced subsets by sampling equal numbers of SLVH-positive and SLVH-negative cases while preserving original train–validation–test proportions for comparison. The final dataset includes 11,190 CXRs (5595 per class) from 6021 patients for training, 658 CXRs (329 per class) from 361 patients for validation, and 534 CXRs from 310 patients for testing. This random sampling was performed with a fixed seed for reproducibility. All images are resized to 256 × 256 and normalized using ImageNet preprocessing statistics. Figure 2 shows the training data distribution for key demographic and anatomical attributes.

### 2.2. SLVH Classification Architectures

We investigate two representative deep learning architectures for direct classification of severe left ventricular hypertrophy (SLVH) from chest radiographs: (i) a ResNet-18 convolutional neural network and (ii) a Vision Transformer (ViT) encoder pretrained using masked autoencoding. These models are selected to contrast convolutional and transformer-based paradigms in terms of spatial feature extraction and global context modeling, respectively.

#### 2.2.1. ResNet-18: Convolutional Baseline

ResNet-18 [13] serves as our convolutional baseline. It comprises a stack of residual blocks that hierarchically extract increasingly abstract visual features by aggregating local patterns through convolutional filters. The model is initialized with weights pretrained on the ImageNet dataset containing 14 million data and image pairs. This initialization aids convergence and facilitates generalization.

For SLVH classification, we fine-tune the entire ResNet-18 model end-to-end using the binary cross-entropy loss. A custom classification head, consisting of two fully connected layers interleaved with ReLU activation and dropout regularization, is appended to the global average pooled feature vector. This head transforms the learned spatial features into a scalar probability representing SLVH likelihood.

#### 2.2.2. Vision Transformer: Masked Autoencoder Pretraining

Our second model employs a foundational Vision Transformer (ViT) encoder, pretrained using the Masked Autoencoder (MAE) framework [14], to capture rich global representations from chest radiographs in a self-supervised manner. Unlike convolutional networks that primarily model local spatial dependencies, ViTs operate on non-overlapping image patches and utilize self-attention mechanisms to learn long-range interactions across the entire image. This is particularly advantageous for cardiothoracic pathologies like SLVH, where diagnostic cues may be spatially diffuse and globally distributed.

The MAE pretraining paradigm is designed to enhance the semantic abstraction capabilities of the encoder. During pretraining, a large fraction (in this case optimal masking ratio is 90%) of input patches are randomly masked, and the model is tasked with reconstructing the missing patches using only the visible subset. The encoder processes the unmasked patches, while a lightweight decoder reconstructs the full image from the encoded tokens. This architecture encourages the encoder to efficiently compress high-level semantic information, as it must infer the global context of the image from sparse visual cues.

In our setup, the ViT encoder is pretrained on a corpus of 300,000 chest radiographs from the MIMIC-CXR [15], NIH ChestX-ray14 [16], and Stanford CheXpert [17] datasets, allowing it to internalize domain-specific structural priors. For downstream SLVH classification, we freeze the pretrained encoder to preserve its learned representations and append a lightweight task-specific MLP head on top of the [CLS] token. The classification head, trained using binary cross-entropy loss, maps the global representation to a probability score indicating SLVH presence.

Freezing the encoder offers several benefits: it reduces computational overhead, prevents catastrophic forgetting of prelearned thoracic features, and isolates the contribution of the classification head in downstream adaptation. Moreover, this setup allows us to evaluate how well the self-supervised ViT embeddings that are learned independently of any diagnostic label encode clinically salient cues relevant to left ventricular hypertrophy.

### 2.3. Expressivity Analysis via Mutual Information Estimation

To quantify the extent to which a model’s internal representations encode clinically relevant information beyond the supervision provided during training, we estimate their *expressivity* using Mutual Information (MI). Expressivity refers to how much internal features retain signal about auxiliary attributes not used as inputs or targets, yet highly relevant to the clinical phenotype. We focus on five such attributes linked to left ventricular hypertrophy: age, sex, interventricular septal diameter (IVSDd), posterior wall diameter (LVPWDd), and left ventricular internal diameter (LVIDd). Analyzing the expressivity of these factors enables post hoc interpretability by revealing the degree to which clinically meaningful associations are implicitly learned. MI quantifies statistical dependence between random variables. Given a learned feature vector $f\in R^{d}$ and attribute *a*, the mutual information I(F;A) measures how much knowledge of one reduces uncertainty about the other. It is formally defined as the Kullback–Leibler divergence between the joint distribution $P_{FA}$ and the product of marginals $P_{F}⊗P_{A}$:

(1) $$I\left(F;A\right)=D_{\mathrm{KL}}\left(P_{FA}\;∥\;P_{F}⊗P_{A}\right),$$

where $D_{\mathrm{KL}}$ quantifies how distinguishable the joint distribution is from one assuming independence.

Since direct estimation of Equation (1) is intractable in high dimensions, we adopt the Donsker–Varadhan (DV) lower bound, implemented via MINE [9]. Here, a neural network $T_{\theta }$ is trained to distinguish between true (joint) and mismatched (marginal) feature–attribute pairs:

(2) $$I_{\theta }\left(F;A\right)\geq E_{P_{FA}}\left[T_{\theta }\left(f,a\right)\right]-\log E_{P_{F}⊗P_{A}}\left[e^{T_{\theta }\left(f,a\right)}\right].$$

This setup effectively implements a “flip-and-check” logic: $T_{\theta }$ learns to output higher values for correct (f,a) pairs and lower scores for mismatched combinations. The better it separates the two distributions, the tighter the bound, indicating stronger dependence.

We estimate the expectations in Equation (2) using a mini-batch of size *b*. For the joint term:

(3) $$E_{P_{FA}}\left[T_{\theta }\left(f,a\right)\right]\approx \frac{1}{b}\sum_{i=1}^{b}T_{\theta }\left(f_{i},a_{i}\right),$$

And for the marginal term:

(4) $$E_{P_{F}⊗P_{A}}\left[e^{T_{\theta }\left(f,a\right)}\right]\approx \frac{1}{b}\sum_{i=1}^{b}e^{T_{\theta }\left(f_{i},a_{j}\right)},\;i\neq j.$$

The final loss minimized is the negative DV bound:

(5) $$L_{\mathrm{MINE}}\left(\theta \right)=-\left(\frac{1}{b}\sum_{i=1}^{b}T_{\theta }\left(f_{i},a_{i}\right)-\log\frac{1}{b}\sum_{i=1}^{b}e^{T_{\theta }\left(f_{i},a_{j}\right)}\right).$$

We implement $T_{\theta }$ as a multi-layer perceptron with two hidden layers and ReLU activations. To stabilize training, we apply exponential moving averaging to the marginal term. MINE is trained separately for each attribute and repeated over 10 random seeds to ensure robustness. Expressivity is evaluated at early, mid, and final layers of both ResNet-18 and ViT classifiers. The overall procedure is outlined in Algorithm 1.
**Algorithm 1** Expressivity computation on learned representations**Require:** 
Layer *L*, set of *n* images *I*, attribute vector $A\in R^{n\times 1}$**Ensure:** 
Expressivity measure1:Initialize E←[]                  ▹ To store expressivity values2:Extract features $F\leftarrow {\left[f_{1},f_{2},\ldots ,f_{n}\right]}^{T}$ from *L* after a particular epoch for all i∈I3:Concatenate the features and attributes: X←[F|A]       ▹ Augmentation step4:**for** iteration=1 to *M* **do**5:      Initialize MINE network $T_{\theta }$ based on the dimensions of X6:      Compute expressivity score: e←MINE(X)7:      Append score: E←E∪{e}8:**end for**9:**return** Expressivity←Average(E)

This expressivity analysis provides a principled lens into the structure of latent representations. A higher estimated MI $I_{\theta }\left(F;A\right)$ implies that the model captures meaningful attribute-relevant information without direct supervision, supporting interpretability and informing downstream fairness or calibration strategies.

### 2.4. Implementation Details

For the convolutional baseline, we use ResNet-18 [13], initialized with ImageNet-pretrained weights. It is fine-tuned end-to-end with an appended classification head comprising two fully connected layers (128 → 64 → 1), each followed by ReLU activation and dropout (p=0.3). Training is performed using the Adam optimizer with a learning rate of $1\times 10^{-4}$ and a weight decay of $1\times 10^{-5}$, optimized via binary cross-entropy loss. For the transformer-based model, we use a ViT encoder pretrained via masked autoencoding (MAE) on around 300,000 images from MIMIC-CXR, Stanford CheXpert, and NIH ChestX-ray14 [14]. The encoder weights remain frozen, and a task-specific MLP head (512 → 128 → 1) is trained on the [CLS] token representation. Optimization uses linear learning rate warmup followed by cosine decay, with early stopping based on validation AUROC.

The MINE network $T_{\theta }$ is implemented as a multilayer perceptron with two hidden layers of sizes 256 and 64, using ELU activations and Xavier initialization. It is trained using the Adam optimizer (learning rate = $1\times 10^{-3}$, batch size = 100). For each attribute, we extract feature vectors $f_{i}$ from the early, mid, and final layers of the classification models, form the feature matrix $F={\left[f_{1},\ldots ,f_{n}\right]}^{T}$, and concatenate it with the corresponding attribute vector $A\in R^{n\times 1}$ to obtain the input matrix X=[F|A]. The expressivity is computed using the MINE objective, and we average the result across M=10 random seeds for robustness.

All training and MI estimation procedures were conducted using NVIDIA A5000 GPUs.

## 3. Results

We demonstrate that severe left ventricular hypertrophy (SLVH) can be directly classified from chest radiographs using deep learning models, without requiring intermediate echocardiographic regressors or demographic covariates. Our approach bypasses multi-stage processing pipelines and enables efficient end-to-end classification. For reference, the current clinical baseline proposed in [6] employs a two-step approach: (i) regression of anatomical markers such as IVSDd from chest X-rays, followed by (ii) diagnostic thresholding. While this method achieves a reasonably high AUROC of 0.79 [95% CI: 0.76–0.81], it suffers from poor class discrimination under severe class imbalance, yielding an AUPRC of just 0.19 [95% CI: 0.15–0.22].

**Figure 3:**
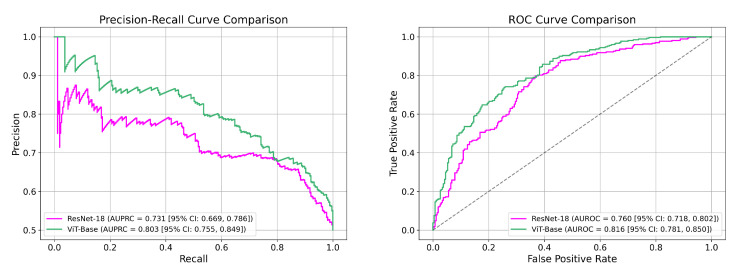
SLVH classification performance shown via (**left**) PR and (**right**) ROC curves for ResNet and ViT backbones.

In contrast, fine-tuning direct classification models on our curated class-balanced dataset yields strong performance across both convolutional and transformer-based architectures, as seen from Table 1 and Figure 3. The ViT-Base achieves the highest classification metrics, with an AUROC of 0.816 [95% CI: 0.781–0.850] and an AUPRC of 0.803 [95% CI: 0.755–0.849], indicating both high sensitivity and reliable precision across decision thresholds. ResNet-18 also performs competitively with an AUROC of 0.760 [95% CI: 0.718–0.802] and AUPRC of 0.731 [95% CI: 0.669–0.786].

### Attribute-Level Expressivity

To probe whether these models learn latent clinical representations despite training only on binary SLVH labels, we apply MINE to quantify the degree to which intermediate features encode five clinically salient attributes: interventricular septal diameter (IVSDd), left ventricular posterior wall diameter (LVPWDd), left ventricular internal diameter (LVIDd), age, and sex. These variables are known factors or diagnostic correlates of SLVH.

**Figure 4:**
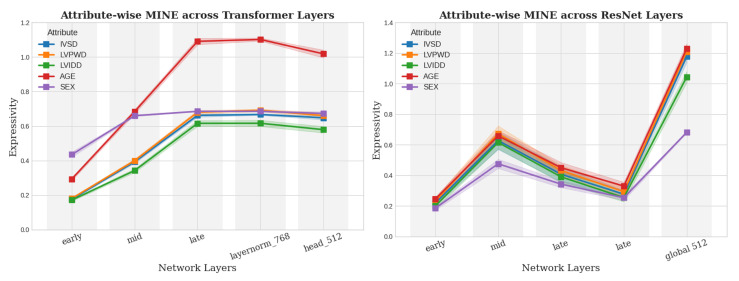
(**Left**) ViT and (**right**) ResNet backbones. The standard deviation of each of the curves is denoted by the shaded region.

Figure 4 summarizes MINE-based expressivity across layers for the ViT and ResNet18 architectures. For ViT, expressivity increases progressively through the transformer blocks and stabilizes in the final layers, suggesting a hierarchical abstraction of clinical attributes across attention layers. In contrast, ResNet18 shows a sharp increase only at the final global average pooling layer, which is consistent with its local to global convolutional design. These architectural differences directly influence where and how clinically relevant signals are encoded.

Notably, age and sex exhibit consistently high expressivity in the final layers of both models, despite not being explicitly provided as input during training. This suggests that the models implicitly infer demographic context from imaging features, likely because these attributes are clinically established contributors to SLVH pathophysiology and diagnosis. While this capability reflects the models’ ability to internalize important diagnostic cues, it also highlights the need for careful evaluation of potential fairness and bias concerns associated with unintended attribute encoding. Among anatomical features, IVSDd and LVPWDd, which are established indicators of myocardial thickening, are consistently encoded with high expressivity, whereas LVIDd shows the lowest expressivity, consistent with its limited diagnostic relevance for hypertrophy. These findings reveal a consistent expressivity ordering for our best model, which further holds for all ablation cases, as described in Section 4:

age>sex>IVSDd>LVPWDd>LVIDd.

This hierarchy reinforces that deep learning classifiers are capable of recovering clinically meaningful structure from chest radiographs using only image-level supervision.

## 4. Discussion

**Figure 5:**
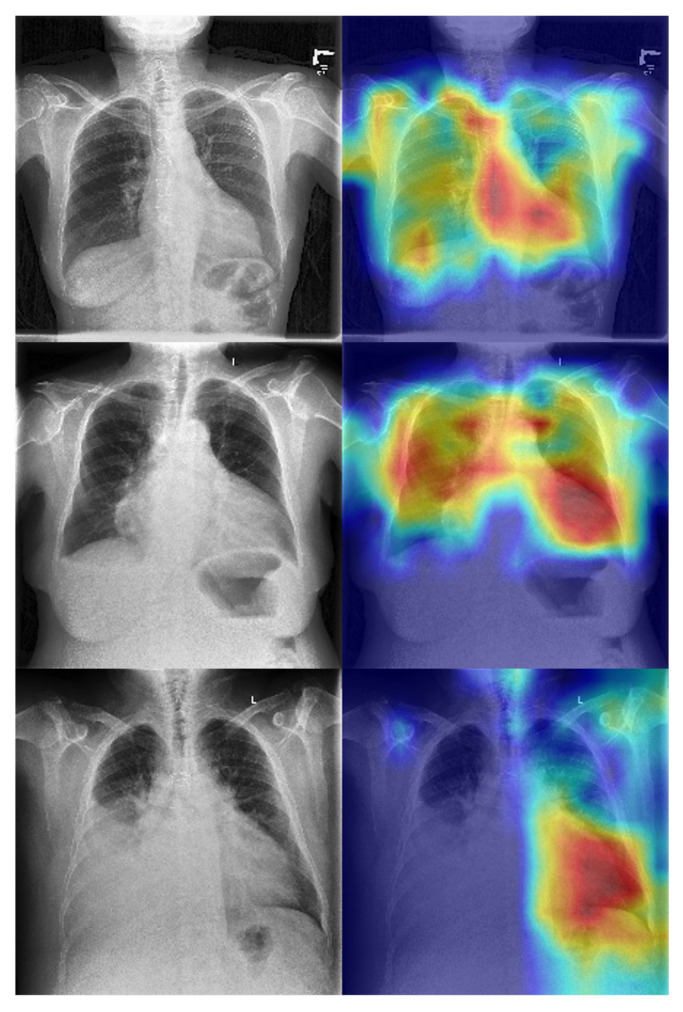
Grad- CAM activation maps for enhanced interpretability on our ViT backbone.

The superior performance of the ViT model is likely driven by its global self-attention mechanism, which enables modeling of long-range anatomical dependencies across the thoracic cavity, a capability that convolutional architectures lack due to their reliance on localized receptive fields. These findings establish that SLVH-relevant cues are sufficiently encoded in chest radiographs to support high-accuracy direct classification when leveraging appropriate architectural priors and pretraining strategies. We show some exemplar GRADCAMs here to further strengthen the interpretability of model decisions in Figure 5. Across multiple examples, we observe that the model consistently attends to central cardiac regions, particularly the mediastinum and left ventricular silhouette, with high-intensity activations (red regions). This localization aligns with the expected anatomical correlate of severe left ventricular hypertrophy, where cardiac enlargement and changes in ventricular wall thickness manifest most prominently in these areas. Importantly, the activations are concentrated around the cardiac contour rather than diffuse pulmonary fields or image borders, suggesting that the network is not relying on spurious features such as rib patterns, background artifacts, or acquisition markers. These interpretability results reinforce the clinical plausibility of the learned features and provide additional transparency. They suggest that the model’s predictions are informed by relevant cardiothoracic structures, in agreement with radiological and cardiological understanding of hypertrophy, thereby increasing confidence in its potential utility.

**Figure 6:**
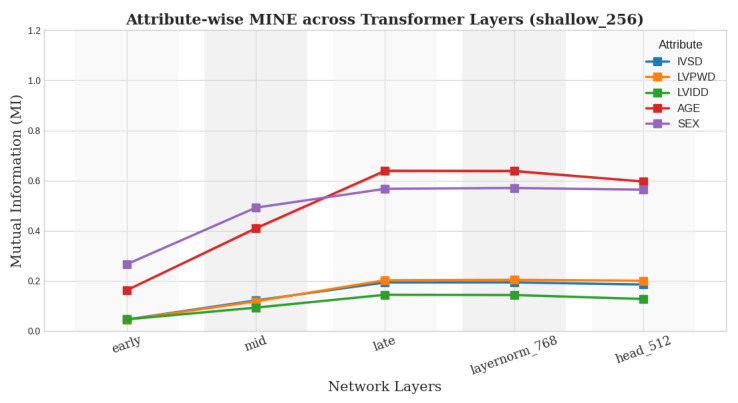
Expressivity trends for ViT using shallower MINE architecture.

**Figure 7:**
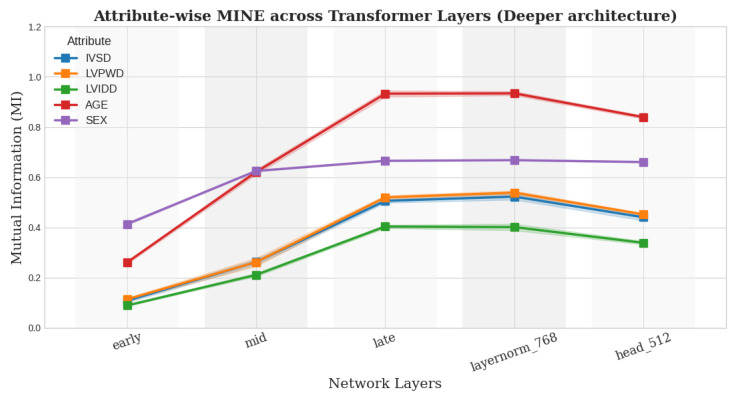
Expressivity trends for ViT using deeper MINE architecture.

For the MINE module, we ablate using different architectural choices. We now evaluated both a shallower design (feature_dim → 256 → 1) and a deeper configuration (feature_dim → 512 → 256 → 64 → 1) in addition to the architecture used previously for our best model, which is the ViT backbone. Importantly, across all tested variants, the relative ordering of attribute expressivity remained the same—age > sex > IVSDd > LVPWDd > LVIDd—which aligns with clinical expectations confirmed by a radiologist as seen from Figure 6 and Figure 7. This consistency underscores the robustness of our conclusions, while also demonstrating that our findings are not sensitive to the precise architectural instantiation of MINE.

## 5. Limitations and Future Work

The major limitation of expressivity is that, being an approximation of mutual information (MI), it depends on entropy, which, in turn, depends on the distribution of attribute labels, which might, in principle, affect absolute value-based comparison between different attributes. This is true for any MI-based technique. However, in our dataset, categorical attributes (sex, SLVH) are well balanced, while continuous attributes (age, IVSDd, LVPWDd, LVIDDd) follow physiologically plausible Gaussian-like distributions without extreme skew. Moreover, our analysis focuses on relative changes across network layers rather than absolute comparisons between unrelated attributes. Thus, the observed trends remain valid, and we additionally verified robustness through ablations. This study highlights the dual importance of (i) foundation models as powerful representation learners, as we can directly detect echocardiographic metrics from chest X-rays, and (ii) MINE-based expressivity analysis as a tool to investigate what these models encode. A natural extension of our framework is to incorporate a broader set of clinical and demographic attributes, such as comorbidities, race, medication history, or lifestyle factors, to obtain a more exhaustive ordering of attribute relevance for detecting cardiac structural abnormalities from chest radiographs. Another promising avenue is attribute suppression, where attributes that are less clinically informative (e.g., LVIDd in the context of hypertrophy, as suggested by both our MINE analysis and clinical guidelines) can be down-weighted or actively suppressed during representation learning. Such targeted interventions may improve the specificity of deep models, mitigate spurious correlations, and bring the learned features closer to clinically actionable biomarkers.

## 6. Conclusions

This study demonstrates that SLVH can be directly and accurately predicted from chest radiographs using modern deep learning architectures, without relying on handcrafted anatomical regressors or auxiliary demographic inputs. We find that a transformer model pretrained on a large corpus of chest radiographs using a self-supervised objective outperforms convolutional models initialized with natural image weights, achieving higher AUROC and AUPRC. This performance gap may reflect the benefits of domain-specific pretraining and the ability of transformers to model long-range anatomical dependencies. Beyond predictive accuracy, we introduce an information-theoretic framework based on MINE to quantify the extent to which clinically meaningful attributes are implicitly encoded across network layers. Our analysis reveals that deep models, even when trained with only binary labels, learn to internalize physiological and demographic correlates of SLVH in a manner that reflects both architectural properties and layer depth. This insight supports the use of post hoc interpretability tools for model auditing, fairness-aware interventions, and more transparent deployment in clinical settings. Our framework provides a foundation for building accurate and interpretable diagnostic models that rely only on images and target labels, and demonstrates how information-theoretic analysis can reveal what clinical attributes are captured within deep learning representations for medical applications.

## Figures and Tables

**Table 1:** Comparison of SLVH classification performance across models. While the baseline model achieves a relatively high AUROC, its AUPRC is limited due to class imbalance. Fine-tuned ResNet-18 improves AUPRC, and the Vision Transformer achieves the best performance on both AUROC and AUPRC (in bold).

Model	AUROC [95% CI]	AUPRC [95% CI]
Baseline (Bhave et al. [6])	0.79 [0.76–0.81]	0.19 [0.15–0.22]
ResNet-18 (fine-tuned)	0.76 [0.72–0.80]	0.73 [0.67–0.79]
ViT-Base (frozen encoder + MLP head)	**0.82** [0.78–0.85]	**0.80** [0.76–0.85]

## Data Availability

The dataset used is public, and the code will be made available upon request to the corresponding author.

## References

[1] Heidenreich P.A., Bozkurt B., Aguilar D., Allen L.A., Byun J.J., Colvin M.M., Deswal A., Drazner M.H., Dunlay S.M., Evers L.R. (2022). 2022 AHA/ACC/HFSA guideline for the management of heart failure: A report of the American College of Cardiology/American Heart Association Joint Committee on Clinical Practice Guidelines. J. Am. Coll. Cardiol..

[2] Cheitlin M.D., Alpert J.S., Armstrong W.F., Aurigemma G.P., Beller G.A., Bierman F.Z., Davidson T.W., Davis J.L., Douglas P.S., Gillam L.D. (1997). ACC/AHA guidelines for the clinical application of echocardiography: A report of the American College of Cardiology/American Heart Association Task Force on Practice Guidelines (Committee on Clinical Application of Echocardiography) developed in collaboration with the American Society of Echocardiography. Circulation.

[3] Heidenreich P.A., Masoudi F.A., Maini B., Chou T.M., Foster E., Schiller N.B., Owens D.K. (1999). Echocardiography in patients with suspected endocarditis: A cost-effectiveness analysis. Am. J. Med..

[4] Khurshid S., Friedman S.F., Pirruccello J.P., Di Achille P., Diamant N., Anderson C.D., Ellinor P.T., Batra P., Ho J.E., Philippakis A.A. (2021). Deep learning to estimate cardiac magnetic resonance–derived left ventricular mass. Cardiovasc. Digit. Health J..

[5] Gurney J. (1995). Why chest radiography became routine. Radiology.

[6] Bhave S., Rodriguez V., Poterucha T., Mutasa S., Aberle D., Capaccione K.M., Chen Y., Dsouza B., Dumeer S., Goldstein J. (2024). Deep learning to detect left ventricular structural abnormalities in chest X-rays. Eur. Heart J..

[7] Lang R.M., Badano L.P., Mor-Avi V., Afilalo J., Armstrong A., Ernande L., Flachskampf F.A., Foster E., Goldstein S.A., Kuznetsova T. (2015). Recommendations for cardiac chamber quantification by echocardiography in adults: An update from the American Society of Echocardiography and the European Association of Cardiovascular Imaging. Eur. Heart J.-Cardiovasc. Imaging.

[8] Bengio Y., Courville A., Vincent P. (2013). Representation learning: A review and new perspectives. IEEE Trans. Pattern Anal. Mach. Intell..

[9] Belghazi M.I., Baratin A., Rajeshwar S., Ozair S., Bengio Y., Courville A., Hjelm D. Mutual information neural estimation. Proceedings of the International Conference on Machine Learning. PMLR.

[10] Donsker M.D., Varadhan S.S. (1975). Asymptotic evaluation of certain Markov process expectations for large time, I. Commun. Pure Appl. Math..

[11] Dhar P., Bansal A., Castillo C.D., Gleason J., Phillips P.J., Chellappa R. (2020). How are attributes expressed in face DCNNs?. Proceedings of the 2020 15th IEEE International Conference on Automatic Face and Gesture Recognition (FG 2020).

[12] Pal B., Huang S., Chellappa R. (2025). A Quantitative Evaluation of the Expressivity of BMI, Pose and Gender in Body Embeddings for Recognition and Identification. arXiv.

[13] He K., Zhang X., Ren S., Sun J. Deep residual learning for image recognition. Proceedings of the IEEE Conference on Computer Vision and Pattern Recognition.

[14] Xiao J., Bai Y., Yuille A., Zhou Z. Delving into masked autoencoders for multi-label thorax disease classification. Proceedings of the IEEE/CVF Winter Conference on Applications of Computer Vision.

[15] Johnson A.E., Pollard T.J., Berkowitz S.J., Greenbaum N.R., Lungren M.P., Deng C.-y., Mark R.G., Horng S. (2019). MIMIC-CXR, a de-identified publicly available database of chest radiographs with free-text reports. Sci. Data.

[16] Wang X., Peng Y., Lu L., Lu Z., Bagheri M., Summers R.M. Chestx-ray8: Hospital-scale chest x-ray database and benchmarks on weakly-supervised classification and localization of common thorax diseases. Proceedings of the IEEE Conference on Computer Vision and Pattern Recognition.

[17] Irvin J., Rajpurkar P., Ko M., Yu Y., Ciurea-Ilcus S., Chute C., Marklund H., Haghgoo B., Ball R., Shpanskaya K. Chexpert: A large chest radiograph dataset with uncertainty labels and expert comparison. Proceedings of the AAAI Conference on Artificial Intelligence.

